# Acute management of pelvic ring injuries: identifying common treatment principles across institutional algorithms

**DOI:** 10.1007/s00590-026-04993-7

**Published:** 2026-10-16

**Authors:** Sascha Halvachizadeh, Christian T. Hübner, Roman Pfeifer, Alona Katzir, Claude Sagi, Nikolaos K. Kanakaris, Peter V. Giannoudis, Ishvinder Grewal, Cyril Mauffrey, Hans-Christoph Pape, Adam J. Starr

**Affiliations:** 1 Department of Trauma, University Hospital of Zurich, Zurich, Switzerland; 2 Department of Orthopaedic Surgery, Parkland Memorial Hospital, Dallas, USA; 3 Department of Orthopaedic Surgery, University of Cincinnati, Cincinnati, USA; 4 Leeds Major Trauma Centre, University of Leeds, Leeds, UK; 5 Department of Orthopedic Surgery, Denver Health Medical Center, Denver, USA

**Keywords:** Pelvic ring injury, Pelvic fracture, Management algorithm, Hemorrhage control, Damage control resuscitation, Systematic review, PRISMA

## Abstract

**Introduction:**

Pelvic ring injuries are associated with substantial morbidity and mortality, particularly in patients presenting with hemodynamic instability and major hemorrhage. Numerous institutions have developed management algorithms for the acute treatment of pelvic ring injuries; however, the common principles underlying these pathways remain unclear.

**Methods:**

A systematic review was conducted in accordance with the PRISMA 2020 guidelines. PubMed/MEDLINE, Embase, and Scopus were searched on January 8, 2026, for peer-reviewed publications published from January 1, 2006, through December 31, 2025 that described algorithms, pathways, or protocols for the acute management of pelvic ring injuries. Two independent reviewers performed study selection and data extraction. Algorithm components related to mechanical stabilization, physiologic assessment, imaging, hemorrhage control, and damage control resuscitation were extracted and compared using descriptive quantitative and qualitative synthesis.

**Results:**

The search identified 328 records that underwent title and abstract screening, and 63 articles were assessed for full-text eligibility. Twenty-five studies were included in the final analysis. All included algorithms (25/25, 100%) used physiology-based decision-making. Early noninvasive mechanical stabilization was specified in 23/25 algorithms (92%); pelvic binders were specified in 17/25 (68%). Angiography with potential angioembolization was included in 19/25 algorithms (76%), exploratory laparotomy in 10/25 (40%), and resuscitative endovascular balloon occlusion of the aorta (REBOA) in 3/25 (12%). Computed tomography was incorporated into 20/25 algorithms (80%), whereas pelvic radiography was included in 18/25 (72%). Fracture morphology was used as a treatment decision point in 4/25 algorithms (16%), and pelvic packing was described in 20/25 publications (80%).

**Conclusion:**

Published algorithms for the acute management of pelvic ring injuries demonstrate substantial consistency despite institutional variation. Early mechanical stabilization, physiologic assessment, damage control resuscitation, radiologic evaluation, and structured hemorrhage-control pathways were identified as the most common components of contemporary management algorithms.

**Supplementary Information:**

The online version contains supplementary material available at https://doi.org/10.1007/s00590-026-04993-7.

## Introduction

Pelvic ring injuries are potentially life-threatening injuries that typically result from high-energy mechanisms such as motor vehicle collisions, falls from height, and crush trauma. Although they account for a small proportion of all fractures, pelvic ring injuries are associated with substantial morbidity and mortality because they frequently occur in the setting of polytrauma and are often accompanied by significant vascular, visceral, and neurological injuries. The incidence of pelvic fracture-associated arterial hemorrhage has been estimated at approximately 1–2 cases per 100,000 population annually, while overall pelvic fractures occur at a rate of approximately 37 per 100,000 individuals per year [1].

Acute hemorrhage following pelvic ring injuries remains the leading cause of early mortality, particularly among hemodynamically unstable patients, with mortality rates exceeding 30% in the absence of structured management protocols [1]. Numerous institutions developed specific algorithms for the treatment of pelvic ring injuries and reported reductions in mortality and morbidity after their introduction [2–5]. These pathways and algorithms reflected local resources, institutional expertise, and the organization of trauma systems [6].

The goal of this study was to identify common treatment principles in published algorithms for the acute management of patients with pelvic ring injuries. We hypothesized that, despite institutional variation, the published algorithms would share core principles involving early mechanical stabilization, physiology-based assessment, damage control resuscitation, diagnostic imaging, and structured hemorrhage control.

## Methods

This systematic review was conducted in accordance with the Preferred Reporting Items for Systematic Reviews and Meta-Analyses (PRISMA) 2020 statement. The protocol was not prospectively registered in PROSPERO or another registry. The objective was to identify and compare published management algorithms for the acute treatment of pelvic ring injuries and to determine common treatment patterns across institutions and trauma systems.

### Search strategy

A systematic literature search was performed in PubMed/MEDLINE, Embase, and Scopus on January 8, 2026, for publications dated January 1, 2006, through December 31, 2025. Controlled vocabulary terms (MeSH in PubMed and Emtree in Embase) and free-text terms for pelvic ring injury, hemorrhage or hemodynamic instability, and management algorithms, pathways, protocols, or guidelines were combined with Boolean operators. Truncation accounted for plural forms and alternate word endings, and database-specific filters excluded inappropriate publication types. The complete database-specific strategies are reported in Supplementary File 1. As a supplementary identification method, the reference lists of all studies selected for full-text assessment and relevant reviews identified during screening were examined manually by two reviewers; potentially eligible records were assessed using the same eligibility criteria. No other websites, registries, organizations, or citation-searching tools were searched.

### Eligibility criteria

Studies were eligible for inclusion if they met the following criteria: (1) were published in a peer-reviewed journal; (2) reported a management algorithm, treatment pathway, protocol, or decision-making framework for the acute treatment of adult patients with pelvic ring injuries; (3) addressed initial assessment, resuscitation, hemorrhage control, stabilization, or definitive management; and (4) were published in English or German within the predefined study period.

Studies were excluded if they: (1) focused exclusively on pediatric populations; (2) addressed isolated surgical techniques without presenting a treatment algorithm; (3) were narrative reviews, editorials, letters, or conference abstracts, or presented expert opinion without a defined management pathway; or (4) did not provide sufficient detail to allow extraction of algorithm components. Because management pathways generally represented expert opinion developed through institutional or consensus processes, pathway inclusion was not interpreted as evidence of comparative effectiveness.

### Study selection

All identified records were imported into a reference management software and duplicates were removed. Two independent reviewers screened titles and abstracts for eligibility. Full-text articles were subsequently assessed for inclusion according to the predefined eligibility criteria. Disagreements were resolved through discussion and consensus. Reasons for exclusion at the full-text stage were documented and reported in the PRISMA flow diagram.

### Data extraction

Data extraction was performed independently by two reviewers using a standardized data collection form. Extracted variables included publication year, country of origin, study design, institution type, patient population, algorithm characteristics, criteria for hemodynamic instability, recommendations for pelvic stabilization, hemorrhage control strategies, use of preperitoneal pelvic packing, angioembolization, resuscitative endovascular balloon occlusion of the aorta (REBOA), external fixation, and multidisciplinary decision-making processes.

### Data synthesis

Given the heterogeneity of study designs and the descriptive nature of the published management algorithms, a meta-analysis of clinical outcomes was not performed. Algorithm components were coded as present or absent and summarized as counts and percentages. A qualitative comparative analysis then categorized the components into initial assessment, hemodynamic evaluation, pelvic stabilization, hemorrhage control, operative intervention, and adjunctive procedures. Common treatment patterns and areas of agreement across algorithms were summarized descriptively.

### Risk of bias assessment

The included publications primarily described institutional protocols, clinical pathways, or management algorithms rather than comparative clinical studies. These pathways constituted expert opinion, with variable methods of consensus development, and a conventional intervention-study risk-of-bias tool was therefore not applicable. Methodological characteristics, the basis for consensus, and reporting completeness were evaluated qualitatively and considered during interpretation.

## Results

**Fig. 1:**
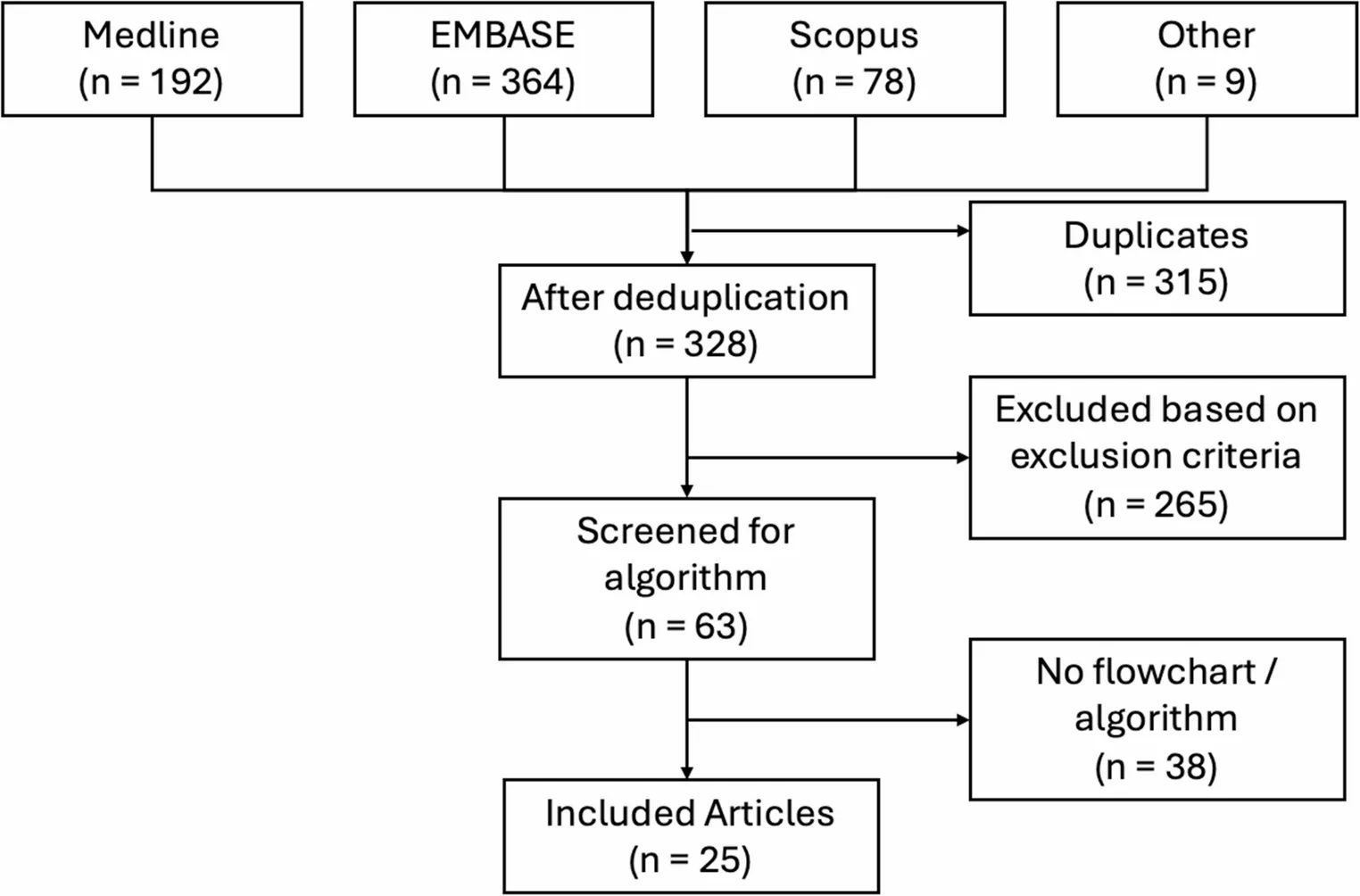
PRISMA flow diagram of study selection. Literature search and screening process for published algorithms addressing the acute management of pelvic ring injury

A total of 643 records were identified: records retrieved from PubMed/MEDLINE, Embase, and Scopus, together with records identified by manual screening of the reference lists of studies assessed in full text and relevant reviews. After removal of 315 duplicates, 328 records underwent title and abstract screening. Application of the predefined eligibility criteria excluded 265 records, leaving 63 reports for full-text assessment. Thirty-eight reports were excluded because they did not provide an eligible management algorithm or flowchart. Consequently, 25 studies [7–31] were included in the final descriptive quantitative and qualitative synthesis (Fig. 1).

### Mortality

Unstable pelvic ring injuries with hemodynamic instability represented a major clinical challenge, with mortality rates historically exceeding 40% and reaching 60% in some series [12, 20, 26]. Several centers reported lower mortality after introducing specialized management protocols [15, 18, 22, 26], but these observational before-and-after findings did not establish that the algorithms independently caused the improvement. One study reported a decrease in 30-day mortality from 63.5% in an earlier phase to 30.6% during a pelvic-packing phase [18]. Another reported a reduction from 47.2% to 23.3% after establishment of a regional trauma center and a new protocol [18]. An 18-year analysis reported that early mortality within 72 h decreased from 27.2% to 16.5% alongside changes in resuscitation and operative care [15] (Table 1). None of the included publications reported formal implementation outcomes such as adoption, protocol adherence, fidelity, or abandonment; the reported associations should therefore not be interpreted as implementation-effectiveness evidence.

**Table 1:** Country and institutional specific mortality rates

Country	Institution / Study	Mortality Rate (%)	Key Cohort Characteristics	Year Published	Study Period (Patients Included)
Switzerland	University Hospitals of Geneva	27.10%	70 hemodynamically unstable Type B/C patients.	2013	Jan 1998 – Dec 2008
	University Hospital Zurich	23.20%	Polytrauma patients.	2021	1996–2012
USA	Denver Health Medical Center	21%	128 patients in refractory shock treated with PPP.	2017	Sep 2004 – Sep 2015
	Presley Regional, Memphis	31%	186 exsanguinating patients; 26% for POD vs. 37% for EPF.	2007	10-year period (ends ~ 2005)
	UT Medical Center, Knoxville	19%	Specifically for open pelvic fractures (52 patients).	2011	Jan 1999 – Dec 2009
	National Trauma Data Bank	12.50%	Overall analysis of 7,473 severe pelvic injuries.	2022	2007–2015
Italy	Niguarda Trauma Center, Milan	21.6%–31.25%	Modern PPP group; historical NO-EPP was 43.2%.	2016	Oct 2002 – Dec 2013
	Papa Giovanni XXIII, Bergamo	30%	30 unstable patients treated with EPP.	2021	Sep 2011 – Dec 2016
Norway	Oslo University Hospital	13%–28%	13% for severe cohort; 28% for exsanguinating EPP cohort.	2007 / 2016	2004–2012
Germany	Goethe University, Frankfurt	12.70%	Overall; increases to 30.0% for Tile C fractures.	2015	Jan 2007 – Dec 2012
Austria	Innsbruck Medical University	33.30%	45 unstable patients; mortality mostly from associated injuries.	2010	Jan 1995 – Dec 2007
France	Grenoble Alps Trauma Center	59%	32 patients with extreme instability requiring REBOA.	2018	Sep 1996 – Aug 2017
Brazil	HC-FMUSP, São Paulo	56.86%	51 patients in shock submitted to EPP.	2022	Oct 2010 – Dec 2016
Hong Kong	Queen Elizabeth Hospital	30.6%–47.2%	30.6% in current PPP phase; 25% specifically for open fractures.	2015	Jan 1996 – Sep 2011
Taiwan	China Medical University	14.3%–16.7%	14.3% for unstable SBP < 90; 16.7% for AE cohort.	2018	Jan 2011 – Dec 2016
South Korea	Regional Trauma Centers	22.6%–23.3%	Recent Korean protocols show mortality around 23%.	2012 / 2019	Jan 2005 – Dec 2016
Israel	Hadassah Hebrew University	26.20%	61 unstable patients undergoing urgent angiography.	2013 / 2019 / 2022	1997–2018
Netherlands	UMC Utrecht (with Harvard)	46%	Unstable cohort	2021	Jan 2001 – Jan 2019
Japan	Teikyo University, Tokyo	13%	HERS Group	2020	Apr 2015 – Dec 2018

All 25 included algorithms (100%) used physiology-based decision-making and emphasized hemodynamic status as a treatment-escalation criterion. Early noninvasive mechanical stabilization was explicitly specified in 23/25 algorithms (92%): pelvic binders in 17/25 (68%), sheets in 3/25 (12%), and other noninvasive devices or terminology in 3/25 (12%). Two algorithms (8%) did not specify a prehospital or initial noninvasive stabilization method (Table 2).

**Table 2:** Emergency intervention

Author	Year	Country	Preclinical mechanical stabilization	Bleeding control	Physiology based algorithm
Tuchayi [10]	2022	USA	Binder	REBOA / Angio	Yes
Jang [11]	2022	South Korea	Binder	Ex Lap	Yes
Fonseca [12]	2022	Brazil	Sheet	Ex Lap	Yes
Magnone [13]	2021	Italy	Binder	Angio	Yes
Frassini [14]	2021	USA / Italy	Binder	Angio	Yes
Tiziani [15]	2020	Switzerland	Binder	Angio	Yes
Hundersmarck [9]	2020	The Netherlands	Binder	Ex Lap	Yes
Ito [16]	2020	Japan	Binder	REBOA	Yes
Frassini [17]	2020	USA	Binder	Angio	Yes
Lee [18]	2019	South Korea	Binder	Ex Lap	Yes
Chou [19]	2019	Taiwan	Not specified	Angio	Yes
Pieper [20]	2018	France	Pelvic belt	Ex Lap Reboa / Angio	Yes
Leung [21]	2018	China	Binder	Angio	Yes
Burlew [32]	2017	USA	Sheet	Angio	Yes
Gaski [22]	2016	Norway	Non invasive stabilization	Ex Lap	Yes
Chiara [8]	2016	Italy	Binder	Angio	Yes
Lustenberger [23]	2015	Germany	Binder	Angio	Yes
Abrassart [24]	2013	Switzerland	Not specified	Ex Lap / Angio	Yes
El-Haj [25]	2013	Israel	Sheet / Binder / Ex Fix	Angio	Yes
Cheng [26]	2012	Hong Kong	Sheet / Binder	Angio	Yes
Black [27]	2011	USA	Binder	Ex Lap / Angio	Yes
Fu [28]	2010	Taiwan	Binder	Ex Lap / Angio	Yes
Jeske [29]	2010	Austria	Wrapper	Ex Lap / Angio	Yes
Tötterman [30]	2007	Norway	Sheet	Angio	Yes
Croce [31]	2007	USA	Pelvic orthotic device	Angio	Yes

*REBOA* Resuscitative Endovascular Occlusion Of the Aorta, *Ex Lap* Exploratory Laparotomy, *Angio* Angiography through interventional radiology

### Preclinical care

Early mechanical stabilization was specified in 23/25 algorithms (92%). Pelvic binders were the most frequently specified method (17/25, 68%), followed by pelvic sheets (3/25, 12%) [7, 12, 30]; one algorithm (4%) used the term “noninvasive stabilization” [22], one (4%) described a pelvic orthotic device [31], and one (4%) described a pelvic wrapper [29]. Two algorithms (2/25, 8%) did not specify an initial noninvasive stabilization method [19, 24].

### Surgical stabilization

Four algorithms (4/25, 16%) incorporated fracture morphology into decision-making. CT imaging was included in 20/25 algorithms (80%) and pelvic radiography in 18/25 (72%), but only four algorithms (16%) translated radiologic findings into fracture-based treatment decisions (Table 3).

**Table 3:** Radiologic assessment

Author	Year	Country	CT Scan	X Ray mentioned	Fracture based algorithm
Tuchayi [10]	2022	USA	Stable /Responder	Yes	No
Jang [11]	2022	South Korea	Stable	no	No
Fonseca [12]	2022	Brazil	Responder	Yes	no
Magnone [13]	2021	Italy	Stable /Responder	Yes	Yes
Frassini [14]	2021	USA / Italy	Stable	no	No
Tiziani [15]	2020	Switzerland	Stable	Yes	No
Hundersmarck [9]	2020	The Netherlands	Stable	Yes	No
Ito [16]	2020	Japan	Stable /Responder	no	No
Frassini [17]	2020	USA	Stable /Responder	Yes	No
Lee [18]	2019	South Korea	Stable	no	No
Chou [19]	2019	Taiwan	Anytime	Yes	No
Pieper [20]	2018	France	Not mentioned	Yes	No
Leung [21]	2018	China	Stable /Responder	Yes	No
Burlew [32]	2017	USA	Stable /Responder	no	No
Gaski [22]	2016	Norway	Not mentioned	no	No
Chiara [8]	2016	Italy	Responder	Yes	No
Lustenberger [23]	2015	Germany	Responder	Yes	Yes
Abrassart [24]	2013	Switzerland	Responder	Yes	No
El-Haj [25]	2013	Israel	Responder	Yes	No
Cheng [26]	2012	Hong Kong	Stable /Responder	Yes	No
Black [27]	2011	USA	Not mentioned	Yes	Yes
Fu [28]	2010	Taiwan	Stable	Yes	No
Jeske [29]	2010	Austria	Responder	Yes	Yes
Tötterman [30]	2007	Norway	Not mentioned	Yes	No
Croce [31]	2007	USA	Not mentioned	Yes	No

Initial operative stabilization was described with an external fixator in 8/25 algorithms (32%) [7, 8, 11, 23, 24, 26, 29, 31]. These publications generally placed external fixation in the initial stabilization phase and often used supra-acetabular pins. Indications or timing included immediately after pelvic radiography [29], persistent physiologic instability despite damage control resuscitation [25], or simultaneous use with preperitoneal pelvic packing [7]. One pathway used the external fixator after removal of the pelvic binder [11].

Some publications described a pelvic C-clamp for immediate stabilization of type C or vertically unstable injuries [21, 23, 24]. They presented the C-clamp as a selective alternative to external fixation, and later series described decreasing use [13, 15].

Two institutions described immediate posterior pelvic-ring screw fixation [15, 25]. One combined sacroiliac screws with an external fixator [25], whereas the other used sacroiliac screws in the emergency setting without an additional external fixator [15].

### Hemorrhage control

Twenty publications (20/25, 80%) described pelvic packing. Reported indications included persistent instability despite mechanical stabilization and damage control resuscitation, particularly when FAST suggested intraperitoneal bleeding requiring laparotomy [7, 24, 29]. One pathway used packing when angiography was unavailable [19], and another linked packing to the need for REBOA [11].

Angiography with potential angioembolization was included in 19/25 algorithms (76%). Six algorithms prioritized identification and treatment of arterial hemorrhage for patients who stabilized sufficiently or met radiologic criteria [10, 15, 16, 19, 25, 29]. Eight used angioembolization after persistent instability or transfusion requirements despite pelvic packing or mechanical stabilization [7–9, 12, 14, 22, 23, 30]. Six publications described angiography as part of a multiprocedural sequence [11, 18, 20, 21, 26, 31], including on-table angiography after external fixation and retroperitoneal packing [21, 26]. These descriptions reflected pathway recommendations rather than comparative proof that angioembolization was universally the treatment of choice.

FAST represented the most common initial triage test in the published pathways. Sixteen algorithms (16/25, 64%) used FAST as a major decision point [11, 12, 14, 16–22, 24, 26, 27, 29, 30], three additionally emphasized repeated assessment of the hemodynamic response [7, 15, 23], and two emphasized CT-based decision-making [15, 16].

Three algorithms (3/25, 12%) explicitly incorporated resuscitative endovascular balloon occlusion of the aorta (REBOA) [10, 16, 20]. Additional publications discussed REBOA as a possible adjunct without embedding it as a defined algorithm node [11–14, 24]. The UK-REBOA randomized clinical trial subsequently found that adding emergency-department REBOA to standard care did not reduce mortality and suggested possible harm; the trial stopped early after a prespecified futility analysis. REBOA should therefore not be presented as routine care or as an established safe intervention. If considered at all, it should be restricted to exceptional circumstances within experienced systems, with recognition of the uncertain benefit and potential harm [33].

Computed tomography (CT) was included in 20 of 25 algorithms (80%), most commonly for hemodynamically stable patients or transient responders following initial resuscitation. Eight algorithms (32%) specifically recommended CT imaging in both stable patients and responders, while six (24%) limited CT evaluation to stable patients and five (20%) to responders. One algorithm (4%) advocated CT imaging regardless of hemodynamic status. Five algorithms (20%) did not explicitly define the role of CT imaging within the treatment pathway. Pelvic radiography was explicitly incorporated into 18 of 25 algorithms (72%).

### The common ground of institutional algorithms

Across the 25 algorithms, physiology-based assessment was present in 25/25 (100%); early noninvasive mechanical stabilization in 23/25 (92%); CT in 20/25 (80%); pelvic radiography in 18/25 (72%); FAST as a major decision point in 16/25 (64%); pelvic packing in 20/25 publications (80%); angiography with potential angioembolization in 19/25 algorithms (76%); exploratory laparotomy in 10/25 (40%); fracture morphology as a treatment decision point in 4/25 (16%); and REBOA as an explicit algorithm node in 3/25 (12%). Damage control resuscitation was a recurring principle, but variable terminology and reporting prevented reliable binary coding across every algorithm. These frequencies describe what the pathways contained, not evidence that any component was effective or implemented consistently (Table 4).

**Table 4:** Frequency of common treatment principles

Decision point	Example	Algorithms/publications, *n*/*N*	Percent
Physiology-based assessment	Hemodynamic status and response to resuscitation	25/25	100%
Early noninvasive mechanical stabilization	Binder, sheet, wrapper, or other device	23/25	92%
Diagnostic imaging framework	CT	20/25	80%
Diagnostic imaging framework	Pelvic radiography	18/25	72%
FAST decision point	Assessment for intraperitoneal bleeding	16/25	64%
Pelvic packing described	Preperitoneal or extraperitoneal packing	20/25	80%
Angiography/angioembolization	Arterial hemorrhage evaluation or control	19/25	76%
Exploratory laparotomy	Operative control of intraperitoneal bleeding	10/25	40%
Fracture morphology decision point	Tile or Young-Burgess pattern	4/25	16%
REBOA explicit algorithm node	Temporary aortic occlusion	3/25	12%

Counts indicate explicit inclusion in the published pathway or, for packing, description in the publication. Damage control resuscitation was not coded because reporting terminology was inconsistent

*CT* computed tomography, *FAST* Focused Assessment with Sonography for Trauma, *REBOA* resuscitative endovascular balloon occlusion of the aorta

### Institution-specific algorithms

Some institutions prioritized early preperitoneal pelvic packing (PPP) for refractory shock [7]. Those authors argued that packing could be performed more rapidly than angiography and could address venous and osseous bleeding [8, 9]. Other pathways, including reports from Taiwan and Japan, prioritized identifying arterial sources with CT or proceeding to angiography and embolization [16, 19, 28].

Some institutional algorithms used radiologic fracture patterns, such as the Tile or Young-Burgess classifications, as decision points for mechanical stabilization and hemostasis. The China Medical University Hospital pathway bypassed CT for hypotensive patients with unstable patterns and directed them to angioembolization or laparotomy; the associated series reported 85.7% overall survival [28]. The Innsbruck pathway applied immediate external stabilization to patients classified as unstable on initial radiography [29]. In a Goethe University series, Tile C injuries were associated with 30% mortality compared with 11.6% for Tile B injuries and with higher transfusion requirements [23]. The Geneva pathway specified a pelvic C-clamp for type C injuries and anterior frames for type B injuries [24]. These observations were descriptive and did not establish comparative effectiveness.

## Discussion

Numerous algorithms for acute pelvic ring injury management were identified, but their recommended sequences varied across institutions and trauma systems. This review compared the published pathways and identified five recurring principles: early mechanical stabilization, repeated physiologic assessment, damage control resuscitation, structured imaging, and escalation pathways for uncontrolled hemorrhage.

- Early noninvasive or minimally invasive mechanical stabilization
- Repeated assessment of the patient’s physiology
- Damage control resuscitation
- A structured imaging framework
- Escalation strategies for uncontrolled bleeding

Noninvasive mechanical stabilization was a central early intervention in the reviewed pathways [34]. The optimal binder position has been debated. A retrospective cohort suggested that application itself might be more important than exact position [35], whereas biomechanical evidence indicated that accurate trochanteric placement improved fracture reduction [36]. In acute care, the intended effects were reduction of pelvic volume, compression of the hemorrhagic space, stabilization of the ring, and support of early clot formation [37]. Pelvic binders were adjuncts rather than definitive treatment [38].

One review reported mortality of approximately 30–40% among hemodynamically unstable patients and emphasized occult retroperitoneal hemorrhage, early shock, and trauma-induced coagulopathy [39]. It proposed pathophysiology-based risk stratification using markers such as lactate, base deficit, coagulation, and temperature. Other publications likewise described assessment of hemorrhage, coagulopathy, soft-tissue injury, and hypothermia [40, 41]. All included algorithms distinguished physiologically unstable from stable or responding patients, although definitions and thresholds varied. Damage control resuscitation approaches also varied across institutions, including transfusion, coagulation, and fluid-resuscitation protocols [42–45].

Imaging recommendations depended on physiology and response to resuscitation. In stable patients or responders, CT was commonly used to assess pelvic-ring and vascular injuries [46]. In unstable patients, pelvic radiography and FAST provided rapid information; FAST was used to identify intraperitoneal bleeding that could prompt laparotomy [47]. Several pathways directed patients with suspected arterial pelvic bleeding toward angiography and embolization [32, 48]. Earlier publications reported potential mortality benefit from preperitoneal packing [49], whereas later observational studies reported possible disadvantages, including venous thromboembolism, reoperation, infection, and neurovascular complications [50, 51]. These nonrandomized findings did not establish the superiority of a packing-first or angiography-first strategy. REBOA was included in only 3/25 algorithms (12%). Importantly, the later UK-REBOA randomized trial found no mortality benefit from adding emergency-department REBOA to standard care and raised concern for possible harm [33]. Accordingly, this review does not advocate routine REBOA use.

### Concepts

The reviewed pathways reflected three overlapping areas of variation: (1) packing-first versus angiography-first hemorrhage control; (2) FAST-based triage versus immediate imaging; and (3) temporary damage-control stabilization versus early definitive fixation. Packing-first pathways prioritized venous and osseous bleeding and emphasized procedural speed [13, 14, 24, 49]. Angiography-first pathways prioritized detection and treatment of arterial bleeding [19, 25, 28]. Other guidance described packing and angiography as complementary rather than mutually exclusive [9]. These were expert-opinion or observational strategies, and the review did not establish superiority of one sequence.

FAST served as a major decision point in 16/25 algorithms (64%). In these pathways, a positive FAST result commonly prompted exploratory laparotomy, whereas a negative result redirected management toward pelvic hemorrhage control [7, 27, 37]. Other algorithms placed less emphasis on FAST and used physiologic response to trigger operative intervention [7, 15].

Mechanical stabilization strategies also varied. Most pathways described immediate binder or sheet application during resuscitation, followed when necessary by temporary external fixation under damage-control principles [14, 21]. The Zurich group instead described a “Safe Definitive Surgery” strategy using early percutaneous sacroiliac screw fixation in selected patients [15]. The publication proposed greater posterior-ring stability and avoidance of external-fixation limitations, but comparative effectiveness was not established [15, 52].

## Limitations

This study had several limitations. The included articles were heterogeneous in study design, institutional setting, trauma-system organization, resources, and definitions of hemodynamic instability. Most publications described expert-opinion pathways with variable consensus methods rather than prospectively validated protocols. The review protocol was not prospectively registered, which increased the risk of selective methodological decisions. Exact archived search histories were unavailable; the database-specific strategies in Supplementary File 1 document the concepts and syntax used for reproducibility but were reconstructed retrospectively. No formal implementation outcomes—such as adoption, adherence, fidelity, or abandonment—were reported, and inclusion of an intervention did not demonstrate implementation or effectiveness. English- and German-language eligibility may have introduced language and publication bias. Geographic representation was also limited: studies from the upper-middle-income economies of Brazil and mainland China were included, but no studies from low-income or lower-middle-income economies were identified. The conclusions may therefore be less transferable to resource-constrained settings. Finally, patient-level outcomes and comparative effectiveness of individual interventions were not assessed.

The algorithms diverged most after initial mechanical stabilization and damage control resuscitation, particularly in hemorrhage-control sequence and timing of definitive fixation. Repeated reassessment of physiologic status was the most consistent cross-cutting decision principle [52].

## Conclusion

Although considerable variation exists among published management algorithms for acute pelvic ring injuries, a high degree of consensus was identified regarding the fundamental principles of care. Early mechanical stabilization, physiological assessment, damage control resuscitation, timely radiologic evaluation, and prompt hemorrhage control represent the core components of contemporary management pathways. These principles may provide a framework for the development and optimization of institution-specific protocols aimed at improving outcomes in this severely injured patient population.

## Supplementary Information

Below is the link to the electronic supplementary material.

Supplementary Material 1

Supplementary Material 2

## Data Availability

This is a systematic review and all data provided are published in scientific journal. The data is referenced correctly and can be reproduced at any time.

## References

[1] de Ridder VA, Whiting PS, Balogh ZJ, Mir HR, Schultz BJ, Routt M (2023) Chip. Pelvic ring injuries: recent advances in diagnosis and treatment. OTA Int 6(3 Suppl):e261. 10.1097/OI90000000000000261. PubMed PMID: 37533441; PubMed Central PMCID: PMC10392441 PMC10392441 37533441

[2] Karadimas EJ, Nicolson T, Kakagia DD, Matthews SJ, Richards PJ, Giannoudis PV (2011) Angiographic embolisation of pelvic ring injuries. Treatment algorithm and review of the literature. Int Orthop (SICOT) 35(9):1381–1390. 10.1007/s00264-011-1271-1 PMC3167455 21584644

[3] Parry JA, Smith WR, Moore EE, Burlew CCC, Mauffrey C (2021) The past, present, and future management of hemodynamic instability in patients with unstable pelvic ring injuries. Injury 52(10):2693–2696. 10.1016/j.injury.2020.02.101 32115215

[4] Pohlemann T, Gänsslen A, Schellwald O, Culemann U, Tscherne H (1996) Outcome after pelvic ring injuries. Injury 27(Suppl 2):B31–38 PubMed PMID: 8915200 8915200

[5] Giannoudis PV, Grotz MRW, Tzioupis C, Dinopoulos H, Wells GE, Bouamra O et al (2007) Prevalence of Pelvic Fractures, Associated Injuries, and Mortality: The United Kingdom Perspective. J Trauma Acute Care Surg 63(4):875. 10.1097/01.ta.0000242259.67486.15 18090020

[6] Cullinane DC, Schiller HJ, Zielinski MD, Bilaniuk JW, Collier BR, Como J et al (2011) Eastern Association for the Surgery of Trauma practice management guidelines for hemorrhage in pelvic fracture–update and systematic review. J Trauma 71(6):1850–1868. 10.1097/TA.0b013e31823dca9a PubMed PMID: 22182895 22182895

[7] Burlew CC, Moore EE, Stahel PF, Geddes AE, Wagenaar AE, Pieracci FM et al (2017) Preperitoneal pelvic packing reduces mortality in patients with life-threatening hemorrhage due to unstable pelvic fractures. J Trauma Acute Care Surg 82(2):233. 10.1097/TA.0000000000001324 PMC5250563 27893645

[8] Chiara O, di Fratta E, Mariani A, Michaela B, Prestini L, Sammartano F et al (2016) Efficacy of extra-peritoneal pelvic packing in hemodynamically unstable pelvic fractures, a Propensity Score Analysis. World J Emerg Surg 11(1):22. 10.1186/s13017-016-0077-2 PMC4888211 27252773

[9] Hundersmarck D, Hietbrink F, Leenen LPH, Heng M (2021) Pelvic packing and angio-embolization after blunt pelvic trauma: a retrospective 18-year analysis. Injury 52(4):946–955. 10.1016/j.injury.2020.11.039 33223257

[10] Tuchayi AM, Nezami N, Zhang Y, Hanna TN, Johnson JO, Newsome J et al (2022) Comparative effectiveness of pelvic arterial embolization versus laparotomy in adults with pelvic injuries: A National Trauma Data Bank analysis. Clin Imaging 86:75–82. 10.1016/j.clinimag.2022.03.008 35367866

[11] Jang JY, Bae KS, Kang BH, Lee GJ (2022) Comparison between external fixation and pelvic binder in patients with pelvic fracture and haemodynamic instability who underwent various haemostatic procedures. Sci Rep 12(1):3664. 10.1038/s41598-022-07694-3 PMC8901771 35256684

[12] Fonseca VC, Menegozzo CAM, Cardoso JMDF, Bernini CO, Utiyama EM, Poggetti RS (2022) Predictive factors of mortality in patients with pelvic fracture and shock submitted to extraperitoneal pelvic packing. Rev Col Bras Cir 49:e20223259. 10.1590/0100-6991e-20223259-en PMC10578839 36197344

[13] Magnone S, Allievi N, Ceresoli M, Coccolini F, Pisano M, Ansaloni L (2021) Prospective validation of a new protocol with preperitoneal pelvic packing as the mainstay for the treatment of hemodynamically unstable pelvic trauma: a 5-year experience. Eur J Trauma Emerg Surg 47(2):499–505. 10.1007/s00068-019-01115-3 30955052

[14] Frassini S, Gupta S, Granieri S, Cimbanassi S, Sammartano F, Scalea TM et al (2021) Emergency Management of Pelvic Bleeding. J Clin Med 10(1):129. 10.3390/jcm10010129 PMC7795542 33401504

[15] Tiziani S, Halvachizadeh S, Knöpfel A, Pfeifer R, Sprengel K, Tarkin I et al (2021) Early fixation strategies for high energy pelvic ring injuries – the Zurich algorithm. Injury 52(10):2712–2718. 10.1016/j.injury.2020.07.036 32736823

[16] Ito K, Nagao T, Tsunoyama T, Kono K, Tomonaga A, Nakazawa K et al (2020) Hybrid emergency room system improves timeliness of angioembolization for pelvic fracture. J Trauma Acute Care Surg 88(2):314. 10.1097/TA.0000000000002544 31804417

[17] Frassini S, Gupta S, Granieri S, Cimbanassi S, Sammartano F, Scalea TM et al (2020) Extraperitoneal packing in unstable blunt pelvic trauma: A single-center study. J Trauma Acute Care Surg 88(5):597. 10.1097/TA.0000000000002618 32317576

[18] Lee MA, Yu B, Lee J, Park JJ, Lee GJ, Choi KK et al (2019) Effects of the establishment of a trauma center and a new protocol on patients with hemodynamically unstable pelvic fractures at a single institution in Korea. Eur J Trauma Emerg Surg 45(2):273–279. 10.1007/s00068-017-0899-y 29344707

[19] Chou CH, Wu YT, Fu CY, Liao CH, Wang SY, Bajani F et al (2019) Hemostasis as soon as possible? The role of the time to angioembolization in the management of pelvic fracture. World J Emerg Surg 14(1):28. 10.1186/s13017-019-0248-z PMC6567387 31210779

[20] Pieper A, Thony F, Brun J, Rodière M, Boussat B, Arvieux C et al (2018) Resuscitative endovascular balloon occlusion of the aorta for pelvic blunt trauma and life-threatening hemorrhage: A 20-year experience in a Level I trauma center. J Trauma Acute Care Surg 84(3):449. 10.1097/TA.0000000000001794 29298239

[21] Leung HCA, Chui KH, Lee KB, Li W (2018) Outcome of Haemodynamically Unstable Open Pelvic Fracture Patients Managed With 3-in-1 Pelvic Damage Control Protocol in a Major Trauma Centre. J Orthop Trauma Rehabilitation 25:62–68. 10.1016/j.jotr.2017.10.002

[22] Gaski IA, Barckman J, Naess PA, Skaga NO, Madsen JE, Kløw NE et al (2016) Reduced need for extraperitoneal pelvic packing for severe pelvic fractures is associated with improved resuscitation strategies. J Trauma Acute Care Surg 81(4):644. 10.1097/TA.0000000000001139 27257711

[23] Lustenberger T, Wutzler S, Störmann P, Laurer H, Marzi I The role of angio-embolization in the acute treatment concept of severe pelvic ring injuries. Injury. 2015;Current Concepts and Innovations in Trauma Care in Germany46:S33–S38. 10.1016/S0020-1383(15)30016-4 26542864

[24] Abrassart S, Stern R, Peter R (2013) Unstable pelvic ring injury with hemodynamic instability: What seems the best procedure choice and sequence in the initial management? Orthop Traumatology: Surg Res 99(2):175–182. 10.1016/j.otsr.2012.12.014 23462306

[25] El-Haj M, Bloom A, Mosheiff R, Liebergall M, Weil YA (2013) Outcome of angiographic embolisation for unstable pelvic ring injuries: Factors predicting success. Injury 44(12):1750–1755. 10.1016/j.injury.2013.05.017 23796438

[26] Cheng M, Cheung MT, Lee KY, Lee KB, Chan SCH, Wu ACY et al (2015) Improvement in institutional protocols leads to decreased mortality in patients with haemodynamically unstable pelvic fractures. Emerg Med J 32(3):214–220. 10.1136/emermed-2012-202009. PubMed PMID: 24327577 24327577

[27] Black EA, Lawson CM, Smith S, Daley BJ (2011) Open pelvic fractures:, the University of Tennessee Medical Center at Knoxville experience over ten years. Iowa Orthop J 31:193–198 PubMed PMID: 22096441; PubMed Central PMCID: PMC3215135 PMC3215135 22096441

[28] Fu CY, Wang YC, Wu SC, Chen RJ, Hsieh CH, Huang HC et al (2012) Angioembolization provides benefits in patients with concomitant unstable pelvic fracture and unstable hemodynamics. Am J Emerg Med 30(1):207–213. 10.1016/j.ajem.2010.11.005 21159470

[29] Jeske HC, Larndorfer R, Krappinger D, Attal R, Klingensmith M, Lottersberger C et al (2010) Management of Hemorrhage in Severe Pelvic Injuries. J Trauma Acute Care Surg 68(2):415. 10.1097/TA.0b013e3181b0d56e 19996797

[30] Tötterman A, Madsen JE, Skaga NO, Røise O (2007) Extraperitoneal Pelvic Packing: A Salvage Procedure to Control Massive Traumatic Pelvic Hemorrhage. J Trauma Acute Care Surg 62(4):843. 10.1097/01.ta.0000221673.98117.c9 17426538

[31] Croce MA, Magnotti LJ, Savage SA, Wood GW, Fabian TC (2007) Emergent Pelvic Fixation in Patients with Exsanguinating Pelvic Fractures. J Am Coll Surg 204(5):935–939. 10.1016/j.jamcollsurg.2007.01.059 17481514

[32] Lindahl J, Gänsslen A (2021) Indirect Hemorrhage Control: Angiography/Embolization (AE). In: Gänsslen A, Lindahl J, Grechenig S, Füchtmeier B, editors. Pelvic Ring Fractures [Internet]. Cham: Springer International Publishing; [cited 2026 Jun 4]. pp. 191–211. Available from: 10.1007/978-3-030-54730-1_15

[33] Jansen JO, Hudson J, Cochran C et al (2023) Emergency Department Resuscitative Endovascular Balloon Occlusion of the Aorta in Trauma Patients With Exsanguinating Hemorrhage: The UK-REBOA Randomized Clinical Trial. JAMA. ;330(19):1862–1871. 10.1001/jama.2023.20850. PMID: 37824132 PMC10570916 37824132

[34] Madsen JE, Flugsrud GB, Hammer N, Puchwein P (2024) Emergency treatment of pelvic ring injuries: state of the art. Arch Orthop Trauma Surg 144(10):4525–4539. 10.1007/s00402-024-05447-7. PubMed PMID: 38970673; PubMed Central PMCID: PMC11576796 PMC11576796 38970673

[35] Reiter A, Strahl A, Kothe S, Pleizier M, Frosch KH, Mader K et al (2024) Does a prehospital applied pelvic binder improve patient survival? Injury 55(4):111392. 10.1016/j.injury.2024.111392. PubMed PMID: 38331685 38331685

[36] Bonner TJ, Eardley WGP, Newell N, Masouros S, Matthews JJ, Gibb I et al (2011) Accurate placement of a pelvic binder improves reduction of unstable fractures of the pelvic ring. J Bone Joint Surg Br 93(11):1524–1528. 10.1302/0301-620X.93B11.27023. PubMed PMID: 22058306 22058306

[37] Costantini TW, Burlew CC, Jenson WR, Pfeifer R, Klingebiel FKL, Kundi R et al (2025) Pelvic fracture bleeding control: What you need to know. J Trauma Acute Care Surg 99(3):319–327. 10.1097/TA.0000000000004609. PubMed PMID: 40170225 40170225

[38] Marchand LS, Sepehri A, Hannan ZD, Zaidi SMR, Bangura AT, Morrison JJ et al (2022) Pelvic Ring Injury Mortality: Are We Getting Better? J Orthop Trauma 36(2):81–86. 10.1097/BOT.0000000000002210. PubMed PMID: 34128497; PubMed Central PMCID: PMC8669037 PMC8669037 34128497

[39] Stahel PF, Ziran N (2024) The pathophysiology of pelvic ring injuries: a review. Patient Saf Surg 18:16. 10.1186/s13037-024-00396-x. PubMed PMID: 38741186; PubMed Central PMCID: PMC11092015 PMC11092015 38741186

[40] Halvachizadeh S, Baradaran L, Cinelli P, Pfeifer R, Sprengel K, Pape HC (2020) How to detect a polytrauma patient at risk of complications: A validation and database analysis of four published scales. PLoS ONE 15(1):e0228082. 10.1371/journal.pone.0228082 PMC6980592 31978109

[41] Pfeifer R, Klingebiel FKL, Halvachizadeh S, Kalbas Y, Pape HC (2023) How to Clear Polytrauma Patients for Fracture Fixation: Results of a systematic review of the literature. Injury 54(2):292–317. 10.1016/j.injury.2022.11.008. PubMed PMID: 36404162 36404162

[42] Khan S, Davenport R, Raza I, Glasgow S, De’Ath HD, Johansson PI et al (2015) Damage control resuscitation using blood component therapy in standard doses has a limited effect on coagulopathy during trauma hemorrhage. Intensive Care Med 41(2):239–247. 10.1007/s00134-014-3584-1. PubMed PMID: 25447807 25447807

[43] Stahel PF, Burlew CC, Moore EE (2017) Current trends in the management of hemodynamically unstable pelvic ring injuries. Curr Opin Crit Care 23(6):511–519. 10.1097/MCC.0000000000000454. PubMed PMID: 29095714 29095714

[44] Spahn DR, Bouillon B, Cerny V, Duranteau J, Filipescu D, Hunt BJ et al (2019) The European guideline on management of major bleeding and coagulopathy following trauma: fifth edition. Crit Care 23(1):98. 10.1186/s13054-019-2347-3. PubMed PMID: 30917843; PubMed Central PMCID: PMC6436241 PMC6436241 30917843

[45] Stahel PF, Moore EE, Schreier SL, Flierl MA, Kashuk JL (2009) Transfusion strategies in postinjury coagulopathy. Curr Opin Anaesthesiol 22(2):289–298. 10.1097/ACO.0b013e32832678ed. PubMed PMID: 19390256 19390256

[46] Leone E, Garipoli A, Ripani U, Lanzetti RM, Spoliti M, Creta D et al (2022) Imaging Review of Pelvic Ring Fractures and Its Complications in High-Energy Trauma. Diagnostics (Basel) 12(2):384. 10.3390/diagnostics12020384. PubMed PMID: 35204475; PubMed Central PMCID: PMC8870907 PMC8870907 35204475

[47] Salcedo ES, Brown IE, Corwin MT, Galante JM Pelvic angioembolization in trauma – Indications and outcomes. Int J Surg 2016;Trauma: Curr Pract Cut Edge Technol 33:231–236. 10.1016/j.ijsu.2016.02.057 26912018

[48] Gänsslen A, Hildebrand F, Pohlemann T (2012) Management of Hemodynamic Unstable Patients in extremis with Pelvic Ring Fractures. ACHOT 79(3):193–202. 10.55095/achot2012/029 22840950

[49] Burlew CC, Moore EE, Stahel PF, Geddes AE, Wagenaar AE, Pieracci FM et al (2017) Preperitoneal pelvic packing reduces mortality in patients with life-threatening hemorrhage due to unstable pelvic fractures. J Trauma Acute Care Surg 82(2):233–42. 10.1097/TA.0000000000001324. PubMed PMID: 27893645; PubMed Central PMCID: PMC5250563 PMC5250563 27893645

[50] Kolitsas A, Williams EC, Lewis MR, Benjamin ER, Demetriades D (2024) Preperitoneal pelvic packing in isolated severe pelvic fractures is associated with higher mortality and venous thromboembolism: A matched-cohort study. Am J Surg 236:115828. 10.1016/j.amjsurg.2024.115828 39059112

[51] Filiberto DM, Fox AD (2016) Preperitoneal pelvic packing: Technique and outcomes. Int J Surg Trauma: Current Practice and Cutting Edge Technology33:222–224. 10.1016/j.ijsu.2016.05.072 27373522

[52] Pape HC, Pfeifer R (2015) Safe definitive orthopaedic surgery (SDS): repeated assessment for tapered application of Early Definitive Care and Damage Control? an inclusive view of recent advances in polytrauma management. Injury 46(1):1–3. 10.1016/j.injury.2014.12.001. PubMed PMID: 25540874 25540874

