# Supplementary material for: Acute management of pelvic ring injuries: identifying common treatment principles across institutional algorithms
Source: Eur J Orthop Surg Traumatol. 2026 Oct 16;36(1):410. doi: 10.1007/s00590-026-04993-7 (PMC13675314; doi:10.1007/s00590-026-04993-7)
Supplement: Supplementary file 1 [file 590_2026_4993_MOESM1_ESM.docx]

**Supplementary File 1
Complete Database Search Strategies**

Search date: January 8, 2026

Coverage: January 1, 2006, through December 31, 2025

Documentation note: The original platform search histories were not archived. The strategies below were reconstructed retrospectively from the prespecified concepts and database syntax to provide a transparent, reproducible record. They should not be interpreted as verbatim exports of the searches run on January 8, 2026.

# PubMed/MEDLINE

(("Pelvic Bones/injuries"[Mesh] OR "Pelvic Fractures"[Mesh] OR pelvic ring injur*[tiab] OR pelvic fracture*[tiab] OR unstable pelvis[tiab]) AND ("Hemorrhage"[Mesh] OR "Shock, Hemorrhagic"[Mesh] OR hemorrhag*[tiab] OR haemorrhag*[tiab] OR hemodynamic*[tiab] OR haemodynamic*[tiab] OR bleeding[tiab] OR shock[tiab]) AND ("Critical Pathways"[Mesh] OR "Practice Guidelines as Topic"[Mesh] OR algorithm*[tiab] OR pathway*[tiab] OR protocol*[tiab] OR guideline*[tiab] OR decision framework*[tiab] OR management strateg*[tiab])) AND ("2006/01/01"[Date - Publication] : "2025/12/31"[Date - Publication]) AND (english[Language] OR german[Language]) NOT (Editorial[Publication Type] OR Letter[Publication Type] OR Comment[Publication Type])

# Embase (Embase.com syntax)

('pelvis fracture'/exp OR 'pelvic ring injury':ti,ab OR 'pelvic ring injuries':ti,ab OR 'pelvic fracture*':ti,ab OR 'unstable pelvis':ti,ab) AND ('bleeding'/exp OR 'hemorrhagic shock'/exp OR hemorrhag*:ti,ab OR haemorrhag*:ti,ab OR hemodynamic*:ti,ab OR haemodynamic*:ti,ab OR bleeding:ti,ab OR shock:ti,ab) AND ('clinical pathway'/exp OR 'practice guideline'/exp OR algorithm*:ti,ab OR pathway*:ti,ab OR protocol*:ti,ab OR guideline*:ti,ab OR 'decision framework*':ti,ab OR 'management strateg*':ti,ab) AND [2006-2025]/py AND ([english]/lim OR [german]/lim) NOT ('conference abstract'/it OR 'conference paper'/it OR 'editorial'/it OR 'letter'/it OR 'note'/it)

# Scopus

TITLE-ABS-KEY(("pelvic ring injur*" OR "pelvic fracture*" OR "unstable pelvis") AND (hemorrhag* OR haemorrhag* OR hemodynamic* OR haemodynamic* OR bleeding OR shock) AND (algorithm* OR pathway* OR protocol* OR guideline* OR "decision framework*" OR "management strateg*")) AND PUBYEAR > 2005 AND PUBYEAR < 2026 AND (LIMIT-TO(LANGUAGE, "English") OR LIMIT-TO(LANGUAGE, "German")) AND NOT (DOCTYPE(ed) OR DOCTYPE(le) OR DOCTYPE(no) OR DOCTYPE(cp))

# Supplementary identification method

Two reviewers manually examined the reference lists of all reports selected for full-text assessment and of relevant reviews encountered during screening. Each title not already present in the deduplicated library was screened, and potentially eligible reports underwent full-text assessment using the same eligibility criteria. No forward-citation search, trial-registry search, website search, organization contact, or author contact was performed.
