# Supplementary material for: Acute management of pelvic ring injuries: identifying common treatment principles across institutional algorithms
Source: Eur J Orthop Surg Traumatol. 2026 Oct 16;36(1):410. doi: 10.1007/s00590-026-04993-7 (PMC13675314; doi:10.1007/s00590-026-04993-7)
Supplement: Supplementary file 2 [file 590_2026_4993_MOESM2_ESM.docx]

**PRISMA 2020 Checklist**

Manuscript: Acute Management of Pelvic Ring Injuries: Identifying Common Treatment Principles Across Institutional Algorithms

| Item | Section/topic | PRISMA requirement | Location in report |
| --- | --- | --- | --- |
| 1 | Title | Identify the report as a systematic review. | Title |
| 2 | Abstract | Structured summary. | Abstract |
| 3 | Rationale | Describe the rationale in context. | Introduction |
| 4 | Objectives | Explicit objective/hypothesis. | Introduction, final paragraph |
| 5 | Eligibility criteria | Specify inclusion/exclusion criteria. | Methods—Eligibility Criteria |
| 6 | Information sources | Specify databases and other sources and last search date. | Methods—Search Strategy |
| 7 | Search strategy | Present full strategies for all databases. | Supplementary File 1 |
| 8 | Selection process | Methods used to decide inclusion. | Methods—Study Selection |
| 9 | Data collection | Methods used to collect data. | Methods—Data Extraction |
| 10a-b | Data items | Outcomes and other variables sought. | Methods—Data Extraction |
| 11 | Risk of bias | Methods used to assess risk of bias. | Methods—Risk of Bias Assessment; not applicable to expert-opinion pathways |
| 12 | Effect measures | Effect measures used. | Not applicable; no comparative effect synthesis |
| 13a-f | Synthesis methods | Eligibility, preparation, tabulation, synthesis, heterogeneity, sensitivity. | Methods—Data Synthesis; descriptive counts/percentages and qualitative synthesis; meta-analysis not performed |
| 14 | Reporting bias | Methods to assess missing-results bias. | Not assessed; no effect estimate synthesis |
| 15 | Certainty assessment | Methods to assess certainty. | Not assessed; no effect estimate synthesis |
| 16a-b | Study selection | Results of search and excluded reports. | Results and Figure 1 |
| 17 | Study characteristics | Characteristics of included studies. | Tables 1-3 |
| 18 | Risk of bias | Risk-of-bias results. | Results/Methods; conventional tool not applicable |
| 19 | Individual study results | Summary statistics/effect estimates. | Not applicable; algorithm components reported in Tables 2-3 |
| 20a-d | Synthesis results | Contributing studies, synthesis, heterogeneity, sensitivity. | Results and Table 4; descriptive synthesis only |
| 21 | Reporting biases | Risk of bias due to missing results. | Not assessed; discussed as publication bias in Limitations |
| 22 | Certainty of evidence | Certainty assessment. | Not assessed; expert-opinion pathways |
| 23a-d | Discussion | Interpretation, limitations of evidence/process, implications. | Discussion and Limitations |
| 24a-c | Registration and protocol | Registration, protocol, amendments. | Methods and Limitations; not prospectively registered; no public protocol or amendment record |
| 25 | Support | Financial/nonfinancial support. | Title page/declarations |
| 26 | Competing interests | Competing interests. | Conflict of interest statement |
| 27 | Availability | Availability of data, code, and materials. | Search strategies supplied; extracted components in Tables 1-4; no analytic code used |

Checklist based on PRISMA 2020. Items not applicable to a descriptive synthesis of expert-opinion pathways are identified explicitly rather than left blank.
